# In-patient neurosurgical tumor treatments for malignant glioma patients in Germany

**DOI:** 10.1007/s11060-024-04784-2

**Published:** 2024-10-10

**Authors:** Marcel A. Kamp, Larissa Fink, Marie-Therese Forster, Carolin Weiss Lucas, Aaron Lawson McLean, Anna Lawson McLean, Christian Freyschlag, Klaus-Peter Stein, Dorothee Wiewrodt, Felix Muehlensiepen, Florian H. Ebner, Marion Rapp, Niklas Thon, Michael Sabel, Nazife Dinc, Christiane von Saß, Marco Stein, Christine Jungk

**Affiliations:** 1 Centre for Palliative and Neuro-palliative Care, Faculty of Health Sciences Brandenburg, Brandenburg Medical School Theodor Fontane, Rüdersdorf bei Berlin, Germany; 2 Department of Neurosurgery, Goethe University Hospital, Frankfurt, Germany; 3 Center of Neuro-Oncology, Department of Neurosurgery, Jena University Hospital, Friedrich-Schiller-University Jena, Jena, Germany; 4 Center for Neurosurgery, Department of General Neurosurgery, University of Cologne, Faculty of Medicine and University Hospital Cologne, Cologne, Germany; 5 Department of Neurosurgery, Medical University Innsbruck, Innsbruck, Austria; 6 Department of Neurosurgery, Otto-Von-Guericke-University, Magdeburg, Germany; 7 Department of Neurosurgery, University Hospital, University Münster, Münster, Germany; 8 Center for Health Service Research Brandenburg, Brandenburg Medical School Theodor Fontane, Rüdersdorf bei Berlin, Germany; 9 Department of Neurosurgery, Alfried Krupp Hospital, Essen, Germany; 10 Centre of Neuro-Oncology, Department of Neurosurgery, Medical Faculty, Heinrich-Heine-University Düsseldorf, Düsseldorf, Germany; 11 Neurosurgical Clinic, University of Munich (LMU), Campus Grosshadern, Munich, Germany; 12 Department of Neurosurgery, Justus-Liebig University, Giessen, Germany; 13 Department of Neurosurgery, University Hospital Heidelberg, Heidelberg, Germany; 14 Department of Neurosurgery, Medical Faculty, Heidelberg University, Heidelberg, Germany

**Keywords:** Malignant glioma, Glioblastoma, Surgery, Mortality, Age, Fluorescence-guided resection, Monitoring, Palliative care

## Abstract

**Objective:**

Treatment for malignant gliomas involves multiple disciplines, including neurosurgery, radiation therapy, medical and neuro-oncology, and palliative medicine, with function-preserving neurosurgical tumor removal being crucial. However, real-world data on hospital cases, treatment types, especially regarding surgical approaches, and the associated complication and mortality rates in Germany are lacking.

**Methods:**

We analyzed data on hospital cases involving malignant gliomas (ICD-10-GM code C71) from the German §21 Hospital Remuneration Act, provided by the Institute for the Hospital Remuneration System (InEK GmbH), from 2019 to 2022. Our focus was on neuro-oncological operations defined by the German Cancer Society (DKG) and included specific operation and procedure (OPS) codes.

**Results:**

From 2019 to 2022, there were 101,192 hospital cases involving malignant gliomas in Germany. Neurosurgical tumor removal was performed in 27,193 cases (26.9%). Microsurgical techniques were used in 95% of surgeries, intraoperative navigation systems in 84%, fluorescence-guided surgeries in 45.6%, and intraoperative neurophysiological monitoring (IONM) in 46.4%. Surgical or medical complications occurred in 2903 cases (10.7%). The hospital mortality rate was 2.7%. Mortality was significantly higher in patients aged 65 and older (Odds ratio 2.9, p < 0.0001), and lower in cases using fluorescence-guided procedures (Odds ratio 0.8, p = 0.015) and IONM (Odds ratio 0.5, p < 0.0001).

**Conclusions:**

Over the course of 4 years, over 100,000 hospital cases involving adult patients diagnosed with malignant gliomas were treated in Germany, with 27,193 cases undergoing tumor removal using various modern surgical techniques. The hospital mortality rate was 2.7%.

**Supplementary Information:**

The online version contains supplementary material available at 10.1007/s11060-024-04784-2.

## Introduction

Malignant glioma, the most common malignant primary brain tumor, significantly affects patient prognosis and quality of life [19]. Left untreated, patients suffering from glioblastoma WHO grade 4 face a prognosis of just 3 months [18]. However, advancements in imaging, histopathology, molecular diagnosis, and therapies have improved outcomes. Neuropathological classification now integrates histopathological and molecular markers, aiding in precise tumor subgroup identification and treatment evaluation [16].

Multidisciplinary teams, including neurologists, neurosurgeons, oncologists, radiation therapists, neuropathologists, radiologists, and palliative care specialists, determine diagnosis and therapy. Surgical resection remains the standard treatment if near-complete tumor removal is feasible without compromising neurological function [12]. Surgical techniques, such as intraoperative tumor visualization using 5-aminolevulinic acid (5-ALA) and intraoperative MRI, have enhanced the extend of resection (EOR) and patient outcome [25–28]. Neuronavigation systems aid in orientation and enable the import of functional and metabolic imaging [1]. Functional cortical and subcortical areas can be localized and preserved using intraoperative neurophysiological monitoring (IONM). Awake procedures can also assess higher cognitive functions and potentially enhance the EOR and patient safety. In addition to surgery, radiotherapy and chemotherapy are part of the standard treatment for malignant gliomas [33]. Post-operative treatment recommendations depend on the tumor's genetic signature, including IDH mutations, 1p/19q co-deletions, and MGMT promoter methylation. Standard treatment for IDH-mutant astrocytomas and oligodendrogliomas, WHO grade 3, comprises resection or biopsy, followed by sequential radio- and chemotherapy [5, 30, 31]. Standard treatment of IDH-wildtype glioblastoma patients under 70 years with a Karnofsky Performance Status (KPS) over 70 includes resection or biopsy, followed by combined chemoradiation [11, 29]. For elderly patients, treatment adapts based on their biological condition and MGMT status, with options including radiotherapy or temozolomide alone [3, 4, 17, 21, 34]. However, malignant gliomas remain incurable, though median survival can exceed 20 months [11]. Therefore, quality of life and early palliative care, recommended within 8 weeks of diagnosis by ASCO guidelines, are essential [9, 33].

A crucial question is how well actual care for neuro-oncological patients meets expectations and demands. In the hospital sector, this involves examining malignant glioma case numbers, treatment types and frequencies, especially surgical removals, and associated complication and mortality rates. Unfortunately, data on these aspects in Germany is lacking. Therefore, we analyzed data on hospital cases involving malignant gliomas (ICD-10-GM code C71) from the German §21 Hospital Remuneration Act, focusing on neurosurgical tumor removal.

## Methods

### Ethics approval, data availability and study design

This study used only publicly available data and followed the ethical principles of the 1964 Helsinki Declaration and its revisions. The Institutional and Local Ethics Committee (study ID 190032024-ANF, Brandenburg Medical School, Germany) approved the study protocol. Data will be available upon reasonable request. The results comply with the STROBE guidelines for epidemiological studies [32].

### Study design, setting and data source

In this cross-sectional study, we evaluated data sourced from the Institute for the Remuneration System in the Hospital Sector (InEK GmbH, Siegburg, Germany) covering the period from 01.01.2019 to 31.12.2022. All hospitals must submit their data, including demographics, diagnoses, and procedures, to InEK GmbH. InEK GmbH, responsible for maintaining the system, aggregates and publishes the data via the InEK Browser, as required by the Hospital Remuneration Act (§21, *Krankenhausentgeltgesetz*).

### Cohort and study size

The InEK data includes hospital in-patient cases but not necessarily the number of patients. It excludes accompanying individuals or cases outside the hospital, but includes pre- and post-hospital services unless subject to separate payment arrangements.

For this analysis, we examined: (1) hospital cases diagnosed with malignant glioma (ICD-10-GM code C71) and (2) patients aged 18 and above. The study size was determined based on an analysis of in-patient cases diagnosed with malignant gliomas between 2019 and 2022.

### Variables and definitions

Data accessed via the InEK browser aggregates datasets with five or more cases, preventing inference of individual cases. We extracted the following data for the specified cohorts from the InEK dataset:

1. Total number of cases for each cohort (entire cohort and those who died during the hospital stay), including cases with C71 as either the primary or secondary diagnosis.
2. Distribution of sex within each cohort.
3. Age distribution across cohorts.
4. Distribution of treating hospitals, categorized by bed size and sponsorship.
5. Count of relevant procedures, identified using the operation and procedure code (OPS).

The definitions of the relevant OPS codes are specified in Table 1 and the ICD-10-GM codes in Table 2.

**Table 1:** Overview about malignant glioma and surgical treatments

	Cases	%	Cases	%
Malignant glioma tumor removal as defined by the OPS codes				
5-015.0, 5-015.1, 5-015.3, 5-015.4, 5-016.0, 5-016.2, 5-016.4, 5-016.6, 5-017.1, 5-035, and 5-075	27,193		736	
Associated OPS codes				

OPS code	Description				
1-20c.0	Navigated transcranial magnetic stimulation (nTMS): in order to identify brain areas for motor function (motor mapping)	381	1.4%		
1-20c.1	Navigated transcranial magnetic stimulation (nTMS): in order to identify brain areas for speech (speech mapping)	192	0.7%		
1-20c.x	Navigated transcranial magnetic stimulation (nTMS): other	67	0.2%		
1-212.0	Invasive intraoperative epilepsy diagnostics: electrocorticography	61	0.2%		
1-212.2	Invasive intraoperative epilepsy diagnostics: electrical stimulation while awake	41	0.2%		
5-012.0	Craniotomy or craniectomy: decompression	194	0.7%	48	6.5%
5-012.1	Craniotomy or craniectomy: drainage of epidural fluid	35	0.1%		
5-012.2	Craniotomy or craniectomy: drainage of an epidural hematoma	153	0.6%		
5-012.3	Craniotomy or craniectomy: drainage of an epidural empyema	89	0.3%		
5-012.6	Craniotomy or craniectomy: reoperation with insertion of a drainage	92	0.3%		
5-013.0	Incision of brain and meninges: drainage of subdural fluid	64	0.2%		
5-013.1	Incision of the brain and meninges: drainage of a subdural hematoma	165	0.6%	11	1.5%
5-013.2	Incision of the brain and meninges: drainage of a subdural empyema	47	0.2%		
5-013.3	Incision of brain and meninges: drainage of intracerebral fluid	77	0.3%		
5-013.40	Incision of the brain and meninges: drainage of an intracerebral hematoma: open surgery	514	1.9%	85	11.5%
5-013.4x	Incision of brain and meninges: drainage of intracerebral hematoma: other	31	0.1%		
5-013.50	Incision of the brain and meninges: drainage of an intracerebral abscess: open surgical	98	0.4%		
5-013.6	Incision of the brain and meninges: removal of an intracerebral foreign body	5	0.0%		
5-983	Reoperation	4553	16.7%	125	17.0%
5-984	Microsurgical technique	25,859	95.1%	700	95.1%
5-988	Use of a navigation system	22,954	84.4%	595	80.8%
5-989	Fluorescence-guided therapy methods	12,400	45.6%	302	41.0%
5-022.00	External ventricular drain	886	3.3%	162	22.0%
5-022.02	External subdural drain	7	> 0.1%		
5-022.0x	Other external CSF drains	57	0.2%		
5-022.20	Ventriculocisternostomy	149	0.5%		
5-023.10	Ventriculoperitoneal shunt	362	1.3%	21	2.9%
5-023.12	Subduroperitoneal shunt	11	> 0.1%		
5-038.0	Spinal CSF drainage	489	1.8%	5	0.7%
6-003.30	Application of carmustine wafer: 4–6 wafer	287	1.1%		
6-003.31	Application of carmustine wafer: 7–9 wafer	179	0.7%		
6-003.32	Application of carmustine wafer: ≥ 10 wafer	13	> 0.1%		
8-925	Intraoperative neurophysiological monitoring	12,606	46.4%	234	31.8%
8-925.00	Intraoperative neurophysiological monitoring: up to 4 h: with stimulation electrodes	531	2.0%		
8-925.01	Intraoperative neurophysiological monitoring: up to 4 h: with evoked potentials (AEP, SEP, MEP, VEP)	3530	13.0%	70	9.5%
8-925.02-04	Intraoperative neurophysiological monitoring: up to 4 h: with cortical electrodes (electrocorticography, phase reversal and/or mapping)	1484	5.5%	10	1.4%
8-925.0x	Intraoperative neurophysiological monitoring: up to 4 h: other	74	0.3%		
8-925.20	Intraoperative neurophysiological monitoring: 4–8 h: with stimulation electrodes	849	3.1%	5	0.7%
8-925.21	Intraoperative neurophysiological monitoring: 4–8 h: with evoked potentials (AEP, SEP, MEP, VEP)	4765	17.5%	137	18.6%
8-925.22-24	Intraoperative neurophysiological monitoring: 4–8 h: with cortical electrodes (electrocorticography, phase reversal and/or mapping)	1038	3.8%	12	1.6%
8-925.2x	Intraoperative neurophysiological monitoring: 4–8 h: other	65	0.2%		
8-925.30	Intraoperative neurophysiological monitoring: 8–12 h: with stimulation electrodes	30	0.1%		
8-925.31	Intraoperative neurophysiological monitoring: 8–12 h: with evoked potentials (AEP, SEP, MEP, VEP)	197	0.7%		
8-925.32-34	Intraoperative neurophysiological monitoring: 8–12 h: with cortical electrodes (electrocorticography, phase reversal and/or mapping)	37	0.1%		
8-925.41	Intraoperative neurophysiological monitoring: > 12 h: with evoked potentials (AEP, SEP, MEP, VEP)	6	> 0.1%

**Table 2:** Overview of selected accompanying diagnoses and complications coded according to ICD-10-GM

	Cases 2019–2022	%	Fatal cases 2019–2022	%
Malignant glioma tumor removal as defined by the OPS codes				
5-015.0, 5-015.1, 5-015.3, 5-015.4, 5-016.0, 5-016.2, 5-016.4, 5-016.6, 5-017.1, 5-035, and 5-075	27,193		736	
Associated OPS codes				

OPS code		Description				
A00-B99		Certain infectious and parasitic diseases	4648	17.1%	382	51.9%
A41		Sepsis	208	0.8%	21	2.9%
D50–D90		Diseases of the blood and blood-forming organs as well as certain disorders involving the immune system	3433	12.6%	304	41.3%
E00–E90		Endocrine, nutritional and metabolic diseases	20,701	76.1%	999	135.7%
F00–F99		Mental and behavioral disorders	7021	25.8%	159	21.6%
	F05	delirium	1544	5.7%	107	14.5%
G00–G99		Diseases of the nervous system				
G00–G09		Inflammatory diseases of the central nervous system	644	2.4%	5	0.7%
	Thereof					
	G00.3	Staphylococcal meningitis	57	0.2%		
	G00.8	Other bacterial meningitis	55	0.2%		
	G00.9	Bacterial meningitis, unspecified	11	0.0%		
	G03.0	Non-suppurative meningitis	39	0.1%		
	G03.8	Meningitis due to other specified causes	52	0.2%		
	G03.9	Meningitis, unspecified	32	0.1%		
	G04.2	Bacterial meningoencephalitis and meningomyelitis, not classified elsewhere	10	0.0%		
	G04.8	Other encephalitis, myelitis and encephalomyelitis	28	0.1%		
	G04.9	Encephalitis, myelitis and encephalomyelitis, unspecified	63	0.2%		
	G06.0	Intracranial abscess and intracranial granuloma	224	0.8%	5	0.7%
	G06.2	Extradural and subdural abscess, unspecified	41	0.2%		
G20–G26		Extrapyramidal diseases and movement disorders	291	1.1%		
G30–G32		Other degenerative diseases of the nervous system	6	0.0%		
G35–G37		Demyelinating diseases of the central nervous system	59	0.2%		
G40–G47		Episodic and paroxysmal diseases of the nervous system	13,179	48.5%	261	35.5%
	Thereof					
	G40	Epilepsy	11,426	42.0%	238	32.3%
	G41	Status epilepticus	299	1.1%	18	2.4%
G50–G59		Diseases of nerves, nerve roots and nerve plexuses	1542	5.7%	51	6.9%
	Thereof					
	G51.0	Facial palsy	1404	5.2%	51	6.9%
C60–C64		Polyneuropathies and other diseases of the peripheral nervous system	241	0.9%	5	0.7%
C80–C83		Cerebral palsy and other paralysis syndromes	8603	31.6%	375	51.0%
	Thereof					
	G81.0	Flaccid hemiparesis and hemiplegia	5226	19.2%	275	37.4%
	G81.1	Spastic hemiparesis and hemiplegia	501	1.8%	16	2.2%
	G81.9	Hemiparesis and hemiplegia, unspecified	949	3.5%	52	7.1%
	G82.03	Flaccid paraparesis and paraplegia: chronic incomplete paraplegia	5	0.0%		
	G82.69	Functional level of spinal cord damage: unspecified	29	0.1%		
	G83.0	Diparesis and diplegia of the upper extremities	35	0.1%		
	G83.1	Monoparesis and monoplegia of a lower extremity	308	1.1%		
	G83.2	Monoparesis and monoplegia of an upper extremity	743	2.7%	7	1.0%
	G83.6	Central facial paresis	680	2.5%	25	3.4%
	G83.8	Other specified paralysis syndromes	127	0.5%		
G90–G99		Other diseases of the nervous system	12,890	47.4%	655	89.0%
	Thereof					
	G91.0	Communicating hydrocephalus	115	0.4%	14	1.9%
	G91.1	Hydrocephalus occlusus	496	1.8%	500	67.9%
	G91.8	Other hydrocephalus	237	0.9%	29	3.9%
	G91.9	Hydrocephalus, unspecified	43	0.2%		
	G93.0	Brain cysts	128	0.5%		
	G93.1	Anoxic brain injury, not classified elsewhere	14	0.1%	7	1.0%
	G93.6	Cerebral edema	8490	31.2%	361	49.0%
	G94.1	Hydrocephalus in neoplasms	219	0.8%	25	3.4%
	G94.2	Hydrocephalus in other diseases classified elsewhere	46	0.2%		
	G96.0	Leakage of cerebrospinal fluid	271	1.0%		
	G96.1	Diseases of the meninges, not classified elsewhere	73	0.3%	5	0.7%
	G96.8	Other specified diseases of the central nervous system	44	0.2%		
	G97.80	Postoperative CSF fistula	735	2.7%	23	3.1%
	G97.88	Other diseases of the nervous system after medical measures	67	0.2%		
H00–H59		Diseases of the eye	4777	17.6%	50	6.8%
	Thereof					
	H02.2	Lagophtalmos	5	0.0%		
	H02.4	Ptosis of the eyelid	69	0.3%		
	H05.2	Exophthalmos	10	0.0%		
	H47.1	Papilla, unspecified	85	0.3%		
	H49.0	Paralysis of the oculomotor nerve (III cranial nerve)	83	0.3%		
	H49.1	Paralysis of the trochlear nerve (IV cranial nerve)	16	0.1%		
	H49.2	Paralysis of the abducens nerve (VI cranial nerve)	84	0.3%		
	H53.2	Diplopia	355	1.3%		
	H53.4	Visual field defects	2201	8.1%	23	3.1%
	H57.0	Pupillary dysfunction	286	1.1%	27	3.7%
H60–H95		Diseases of the ear and mastoid process	438	1.6%		
I00–I99		Diseases of the circulatory system	21,912	80.6%	1075	146.1%
	Thereof					
	I20–I25	Ischemic heart disease	1580	5.8%	43	5.8%
	I25.1	Atherosclerotic heart disease	1006	3.7%	30	4.1%
	I25.2	Old myocardial infarction	356	1.3%	13	1.8%
	I26	Pulmonary embolism	374	1.4%	73	9.9%
	I60.8	Other subarachnoid hemorrhage	61	0.2%	7	1.0%
	I61.0	Intracerebral hemorrhage into the cerebral hemisphere, subcortical	335	1.2%	49	6.7%
	I61.1	Intracerebral hemorrhage into the cerebral hemisphere, cortical	59	0.2%		
	I61.2	Intracerebral hemorrhage into the cerebral hemisphere, unspecified	58	0.2%		
	I61.4	Intracerebral hemorrhage into the cerebellum	44	0.2%		
	I61.5	Intracerebral intraventricular hemorrhage	114	0.4%	33	4.5%
	I61.6	Intracerebral hemorrhage at multiple locations	30	0.1%		
	I61.8	Other intracerebral hemorrhage	203	0.7%	30	4.1%
	I61.9	Intracerebral hemorrhage, unspecified	41	0.2%		
	I62.00	Non-traumatic subdural hemorrhage: acute	109	0.4%	10	1.4%
	I62.01	Non-traumatic subdural hemorrhage: subacute	38	0.1%		
	I62.02	Non-traumatic subdural hemorrhage: chronic	47	0.2%		
	I62.09	Non-traumatic subdural hemorrhage: unspecified	31	0.1%		
	I62.1	Nontraumatic extradural hemorrhage	107	0.4%		
	I62.9	Intracranial hemorrhage (non-traumatic), unspecified	5	0.0%		
	I63.3	Cerebral infarction due to thrombosis of cerebral arteries	72	0.3%		
	I63.4	Cerebral infarction due to embolism of cerebral arteries	59	0.2%		
	I63.5	Cerebral infarction due to unspecified occlusion or stenosis of cerebral arteries	248	0.9%	42	5.7%
	I63.8	Other cerebral infarction	422	1.6%	49	6.7%
	I63.9	Cerebral infarction, unspecified	42	0.2%		
	I65.0	Occlusion and stenosis of the vertebral artery	6	0.0%		
	I65.2	Occlusion and stenosis of the carotid artery	90	0.3%		
	I67.10	Cerebral aneurysm (acquired)	55	0.2%		
	I80	Thrombosis, phlebitis and thrombophlebitis	245	0.9%		
J00–J99		Diseases of the respiratory system	5901	21.7%	783	106.4%
K00–K93		Diseases of the digestive system	3242	11.9%		
L00–L99		Diseases of the skin and subcutaneous tissue	531	2.0%		
	Thereof					
	L89.0	1st Degree decubitus ulcer	64	0.2%		
	L89.1	2nd Degree decubitus ulcer	167	0.6%	15	2.0%
	L89.2	3rd Degree pressure ulcer	23	0.1%		
M00–M99		Diseases of the musculoskeletal system and connective tissue	751	2.8%		
N00–N99		Diseases of the genitourinary system	4715	17.3%	306	41.6%
R00–R99		Symptoms and abnormal clinical and laboratory findings not classified elsewhere	32,824	120.7%	1644	223.4%
	Thereof					
	R20.1	Hyperesthesia of the skin	657	2.4%	6	0.8%
	R20.2	Paresthesia of the skin	185	0.7%		
	R25.2	Cramps and spasms of the muscles	29	0.1%		
	R26.0	Ataxic gait	302	1.1%		
	R26.2	Difficulty walking, not classified elsewhere	76	0.3%		
	R26.3	Immobility	384	1.4%	48	6.5%
	R40.0	Somnolence	907	3.3%	122	16.6%
	R40.1	Sopor	178	0.7%	56	7.6%
	R40.2	Coma, unspecified	150	0.6%	68	9.2%
	R41.0	Disorientation disorder, unspecified	1353	5.0%	71	9.6%
	R41.2	Retrograde amnesia	55	0.2%		
	R41.3	Other amnesia	237	0.9%		
	R41.8	Other and unspecified symptoms affecting recognition and consciousness	171	0.6%		
	R42	Dizziness and staggering	1057	3.9%	32	4.3%
	R43.0	Anosmia	10	0.0%		
	R47.0	Dysphasia and aphasia	5782	21.3%	201	27.3%
	R47.1	Dysarthria and anarthria	1508	5.5%	76	10.3%
	R47.8	Other and unspecified speech and language disorders	396	1.5%	17	2.3%
	R48.0	Dyslexia and alexia	51	0.2%		
	R51	Headache	2411	8.9%	56	7.6%
	R52.0	Acute pain	24	0.1%		
	R52.1	Chronic uncontrollable pain	98	0.4%	5	0.7%
	R52.2	Other chronic pain	136	0.5%	5	0.7%
	R52.9	Pain, unspecified	14	0.1%		
S00–T98		Injuries, poisoning and certain other consequences of external causes	3862	14.2%	168	22.8%
	Thereof					
	T80–T88	Complications of surgical procedures and medical treatment, not classified elsewhere	2903	10.7%	163	22.1%
	T81.0	Bleeding and hematoma as a complication of surgery, not classified elsewhere	972	3.6%	123	16.7%
	T81.1	Shock during or as a result of a procedure, not classified elsewhere	26	0.1%		
	T81.2	Accidental puncture or laceration during a procedure, not classified elsewhere	93	0.3%		
	T81.3	Opening a surgical wound, not classified elsewhere	172	0.6%		
	T81.4	Infection following a procedure, not classified elsewhere	253	0.9%	10	1.4%
	T81.7	Vascular complications after surgery, not classified elsewhere	16	0.1%		
	T81.8	Other complications of procedures, not classified elsewhere	255	0.9%	13	1.8%
	T85.0	Mechanical complication caused by a ventricular intracranial shunt	44	0.2%		
	T85.6	Mechanical complication caused by other specified internal prostheses, implants or transplants	16	0.1%		
	T85.72	Infection and inflammatory reaction caused by internal prostheses, implants or transplants in the nervous system	31	0.1%		
	T88.4	Unsuccessful or difficult intubation	97	0.4%		
	T88.5	Other complications resulting from anesthesia	67	0.2%		
	T88.7	Unspecified undesirable side effect of a medicine or drug	35	0.1%		
	T88.8	Other specified complications of surgical procedures and medical treatment, not classified elsewhere	10	0.0%		

The ICD-10-GM code C71 refers to malignant neoplasms of the brain but does not specify which neoplasms are classified as malignant based on the integrated pathological diagnosis. We considered hospital cases with C71 as either the primary or secondary diagnosis. To calculate the number of cases, we retrieved counts of patients with C71 as either the primary or secondary diagnosis and subtracted double-coded cases. We also separately extracted data for patients who died in the hospital (discharge reason 09: death).

For defining a surgical neuro-oncological tumor resection, we adhered to the definition provided by the German Cancer Society (Deutsche Krebsgesellschaft; DKG). According to this definition, a surgical neuro-oncological tumor resection is determined by the OPS codes 5-015.0, 5-015.1, 5-015.3, 5-015.4, 5-016.0, 5-016.2, 5-016.4, 5-016.6, 5-017.1, 5-035, and 5-075.

### Statistics

Data were retrieved from the InEK data browser and organized using Microsoft Excel for Mac (Version 16.78, Microsoft Corporation, Redmond, Washington, USA). Statistical analyses and graphing were conducted using GraphPad Prism 9 for macOS (Version 9.5.0, GraphPad Software, Inc., La Jolla, USA).

Descriptive statistics were utilized to calculate the percentage of patients who passed away, as well as those who received treatment, Pearson's χ^2^ test was employed to examine differences in distribution of the use of operation techniques in different subgroups [22, 23].

A two-sided significance level of α = 0.05 was applied, and a Bonferroni-procedure was implemented to adjust for multiple comparisons, with n = 3 [3]. Therefore, the adjusted significance level was 0.017. All statistical correlations investigated are described in the results section.

## Results

### Baseline characteristics of patient cohort

**Fig. 1:**
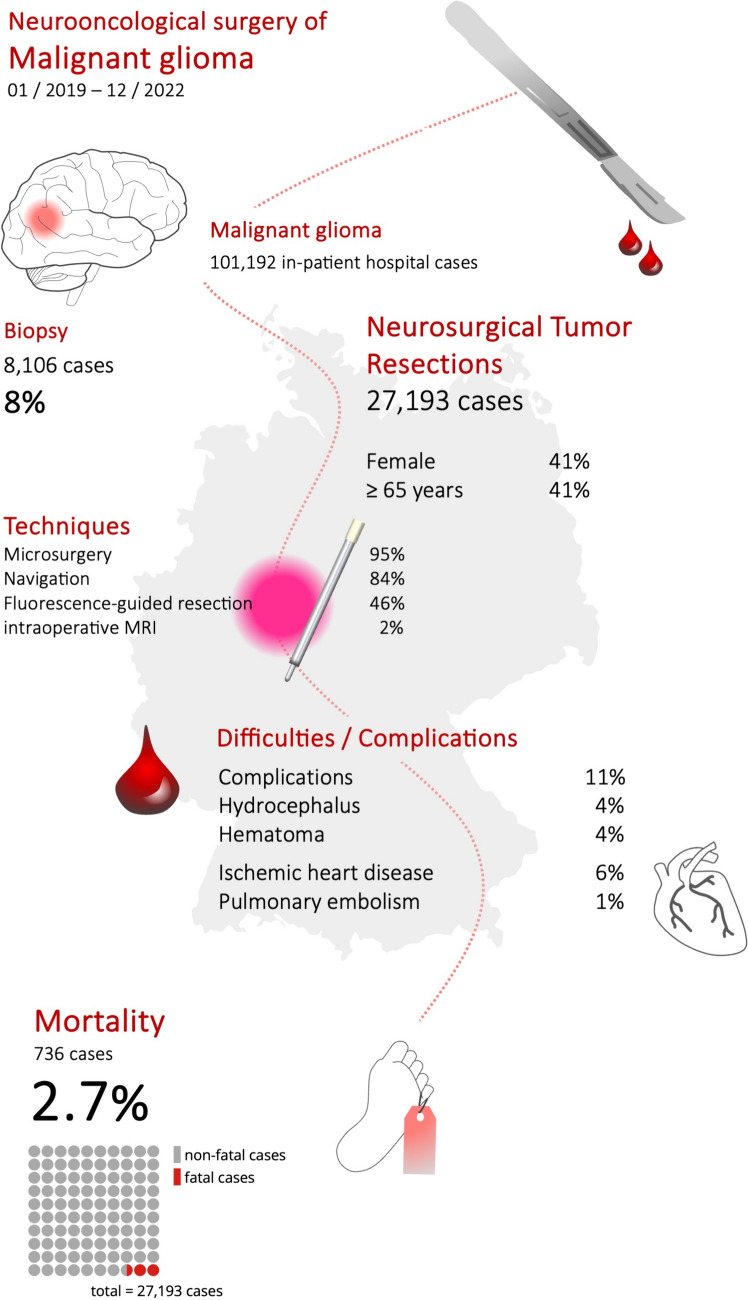
Summary of results

**Fig. 2:**
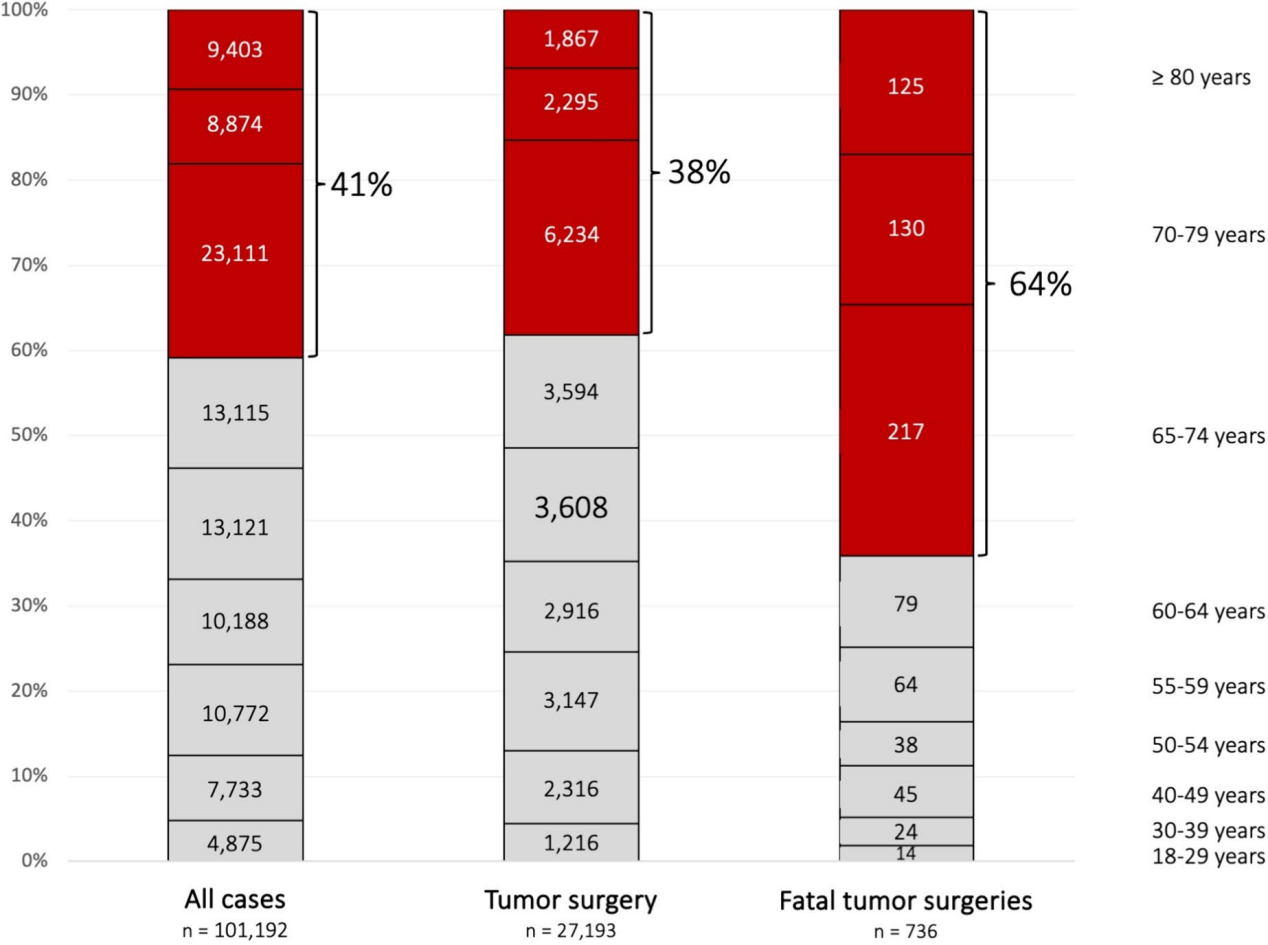
Number of hospital cases sorted by age of patients with malignant gliomas. Figure illustrates both the relative and absolute frequency of hospitalizations of patients diagnosed with malignant gliomas. The number in each column represents the absolute count of cases for that specific age group, with its height indicating the relative frequency. The left column represents the frequency of hospital cases for all patients with malignant gliomas, the middle column depicts hospital cases of patients who underwent tumor resection, and the right column displays the age distribution of patients who passed away during hospitalization for tumor resection. The intervals between the age groups in the figure are not uniform

From 01/2019 to 12/2022, German hospitals handled 101,192 in-patient cases involving adult patients diagnosed with malignant glioma. Malignant glioma was the primary diagnosis in 78,898 cases and a secondary diagnosis in 44,139 cases. 42,733 Cases (42.2%) involved female patients. Patients aged 65 years or older accounted for 41,388 cases (40.9%, Figs. 1, 2).

Most treatments occurred at public hospitals with over 1000 beds (39,424 cases, 39%), followed by public hospitals with 800 to 999 beds (8313 cases, 8.2%).

### Overview of neurosurgical procedures

In-patient biopsies were performed in 8102/101,192 in-patient glioma cases (8%), including 2894 open biopsies (35.7%) and 5208 stereotactic biopsies (64.3%).

**Fig. 3:**
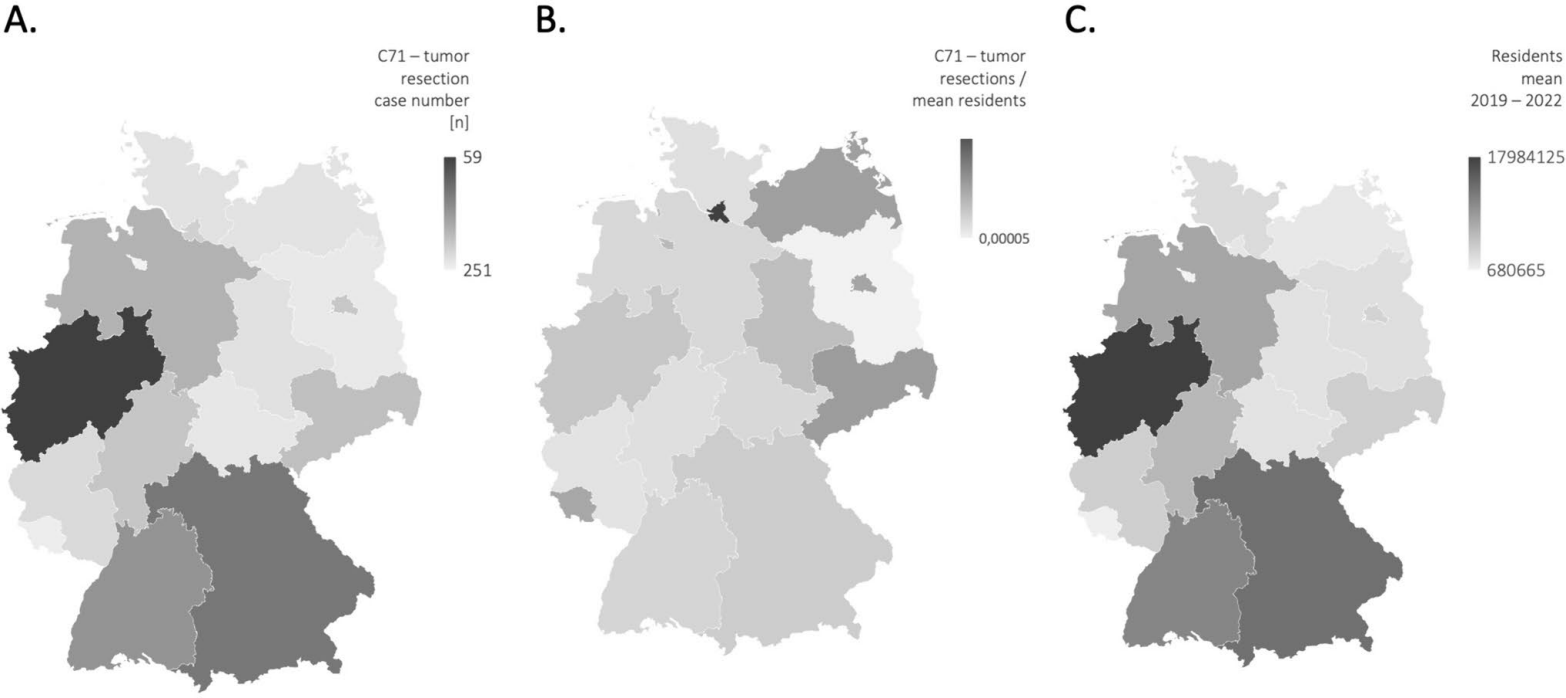
Case numbers for surgical removal of malignant gliomas across individual federal states from 2019 to 2022

According to the German Cancer Society’s definition, surgical removal occurred in 27,193 cases, representing 26.9% of all hospital cases involving malignant glioma from 2019 to 2022. During the SARS-CoV-2 pandemic years of 2020 and 2021, case numbers were slightly higher (Supplementary Fig. 1). Figure 3 illustrates the total case numbers for surgical removal of malignant gliomas across individual federal states from 2019 to 2022 (Supplementary Table 1).

### Detailed analysis of neurosurgical tumor resections

**Fig. 4:**
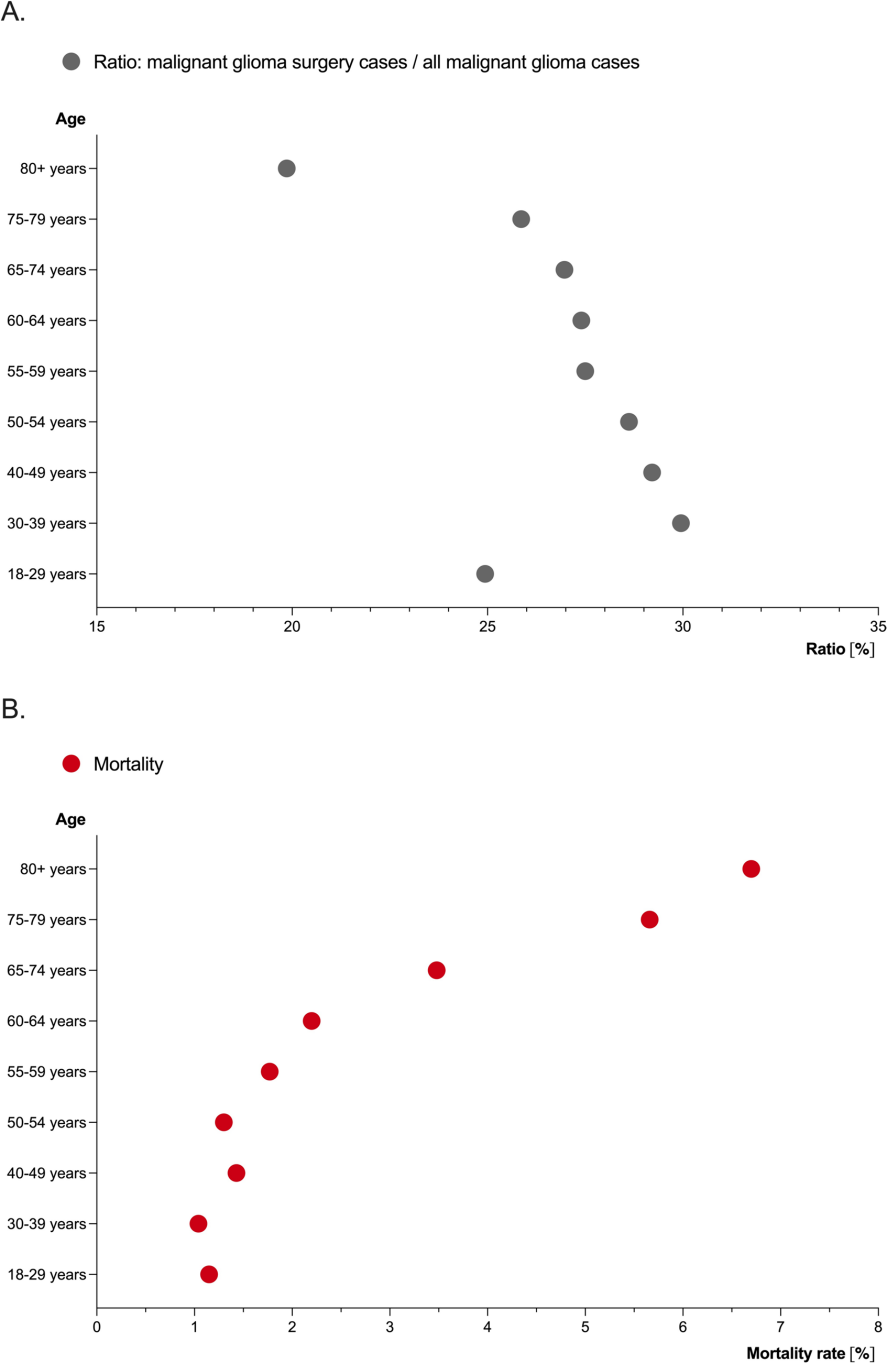
Proportion of tumor resections and mortality per age group. Figure illustrates the proportion of hospital cases with resections of malignant gliomas (**A**) and their corresponding in-patient mortality rate (**B**) across different age groups

A detailed analysis of the surgical techniques used in the 27,193 cases of tumor removal is warranted (Table 1). Among these cases, 11,169 involved female patients (41.1%). Notably, the majority of patients were aged 65 years or older (10,396 cases, 41%, Fig. 4). A total of 4553 cases (16.7%) were coded as reoperation, and in 72 cases (2.6%), operations were prematurely terminated. Dural reconstruction (duraplasty) was performed in 19,750 cases (72.6%).

**Fig. 5:**
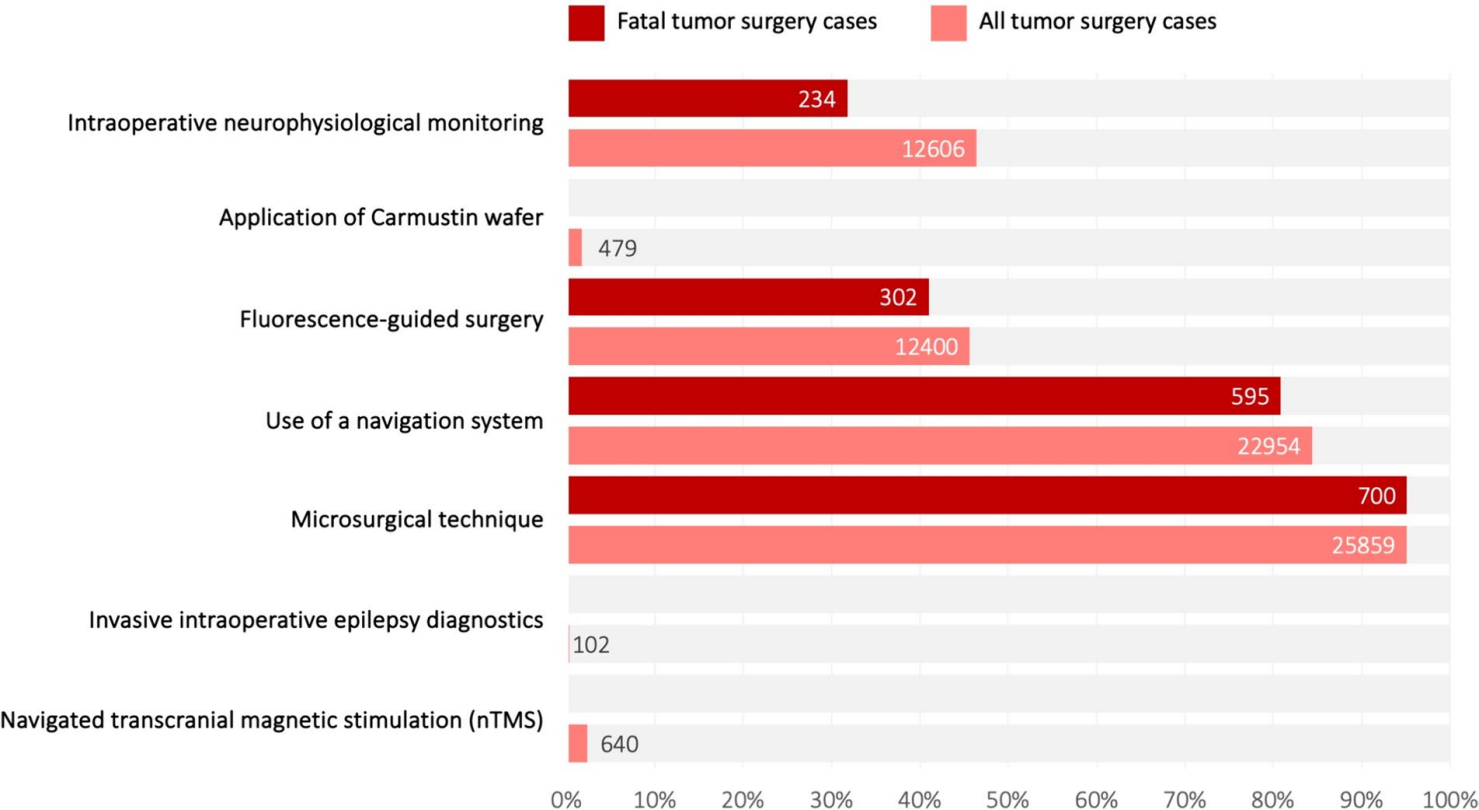
Surgical techniques used. Figure presents an overview of the frequency of resection techniques utilized in hospital cases involving resections of malignant gliomas. Each column displays the absolute count of cases, with its height reflecting the relative frequency. Light red bars represent data for the entire cohort, while dark red bars indicate data for deceased patients

Neurosurgeons routinely employed microsurgical techniques in 25,859 cases (95%), with intraoperative navigation systems utilized in 22,954 cases (84.4%, Fig. 5). Fluorescence-guided resection methods were noted in a total of 12,400 cases (45.6%; OPS code 5-989). However, the specific fluorophore used, whether 5-ALA or fluorescein, could not be discerned from the data. We determined the rate of intraoperative MRIs by combining the OPS codes 3-800 and 3-992. This combination was found in 499 cases (1.8%). Over the 4 years, carmustine wafers were intraoperatively implanted in 479 cases (1.7%). ´

IONM accompanied tumor removal in 12,606 cases (46.4%). Monitoring durations varied, with less than 4 h in 5619 cases (44.6% of IONM cases), between 4 and 8 h in 6717 cases (53.3%), and more than 8 h in 270 cases (2.1%). Evoked potentials (AEPs, SEPs, MEPs, VEPs) were employed in 8498 cases (31.3% of all tumor removals), while electrocorticography, phase reversal, and/or mapping were utilized in 2995 cases (11%). Additionally, intraoperative invasive epilepsy diagnostics was applied in 102 cases (0.4%), and preoperative navigated transcranial magnetic stimulation (nTMS) was documented in 640 cases (2.3%).

### Complications and accompanying symptoms during hospitalization after tumor resections

Table 2 summarizes complications and accompanying symptoms in 27,193 tumor resection cases. Surgical or medical complications not otherwise classified were coded in 2903 cases (10.7%; for a comparison with hospital cases involving patients with malignant gliomas treated with radiotherapy or medical tumor therapy see Supplementary Table 2). Postoperative hematoma occurred in 972 cases (3.6%), with 710 cases (2.6%) requiring surgical evacuation. Hydrocephalus was present in 1156 cases (4.3%), with 886 cases (3.3%) needing intraventricular drainage and 362 cases (1.3%) requiring ventriculoperitoneal shunts. Anesthesiologic complications occurred in 67 cases (0.2%), and difficult intubation in 97 cases (0.4%). Ischemic heart disease affected 1580 patients (5.8%), and pulmonary embolism was reported in 374 patients (1.4%).

### In-patient mortality following neurosurgical tumor resections

Among 27,193 hospital cases involving tumor resection for malignant glioma, 736 patients (2.7%) died during the same hospital stay. Of these, 285 (38.7%) were female. The age distribution of deceased patients is shown in Fig. 1, with surgical techniques detailed in Table 2. Patients aged 65 and older had a significantly higher mortality rate (Odds ratio 2.9; 95% CI 2.5–3.4; p < 0.0001), increasing from about 1% in younger age groups (18–39 years) to nearly 7% in those ≥ 80 years old. Fluorescence-guided resection (Odds ratio 0.83; 95% CI 0.72–0.96; p = 0.015) and IONM (Odds ratio 0.54; 95% CI 0.46–0.63; p < 0.0001) were less frequently used in fatal cases. Mortality did not increase during the SARS-CoV-2 pandemic years of 2020 and 2021.

### Adjuvant antitumor treatments and palliative care

Radiation therapy was administered during in-patient stays in 14,686 cases (14.5% of all malignant glioma), predominantly utilizing intensity-modulated radiation therapy with a linear accelerator (11,140 cases; 11%). Additionally, stereotactic irradiation was carried out in 727 cases (0.7%). Chemotherapy during hospitalization was coded in 4032 cases (4%).

Among 101,192 in-patient cases of adult malignant glioma, 10,592 (10.5%) involved complex or specialized palliative care [10]. In 1303 of the 27,193 hospital cases involving tumor resection (4.8%), complex or specialized palliative care was provided. Specifically, 301 cases involved complex palliative care, 213 cases involved specialized palliative care on a palliative care unit, and 789 cases involved care provided by a consultation service.

## Discussion

We analyzed German in-patient data on 101,192 cases involving adult patients diagnosed with malignant gliomas (ICD code C71) from 2019 to 2022, providing insights into real-world scenarios. These are the main findings:

1. Surgical removal occurred in 27,193 cases (26.9% of all hospital cases). Microsurgical techniques were used in 95%, intraoperative navigation in 84%, fluorescence-guided surgeries in 45.6%, and IONM in 46.4%.
2. Surgical or medical complications not otherwise classified were coded in 10.7% of tumor removal cases.
3. Among the 27,193 hospital cases involving tumor resections, 736 patients passed away during the same hospital stay, resulting in a hospital mortality rate of 2.7%. The proportion of patients aged 65 years or older was higher and fluorescence-guided resection techniques and IONM were less often utilized in fatal surgical cases.

101,192 In-patient cases involved adult patients with malignant gliomas in Germany in a 4-year period, accounting for 0.16% of all hospitalizations. Neurosurgical tumor resections were performed in about 27% of these cases. In relation to the average population, Hamburg and Berlin exhibit notably high rates of surgical resections (Abb. 3). This might be attributed to their neuro-oncological centers, which attract a significant number of patients from neighboring rural areas.

Microsurgical techniques were used in 95% of these surgeries, with neuronavigation systems applied in 85%. Intraoperative MRI was employed in 1.8%, and fluorescence-guided resection was performed in 45.6% of tumor removal cases. While the specific fluorophore (5-ALA or fluorescein) was not identified, many surgeries likely used 5-ALA. Studies have shown that 5-ALA-guided resection and intraoperative MRI improve the EOR, with 5-ALA also enhancing patient outcome [25–28]. The low rate of tumor visualization techniques, used in less than 50% of cases, may result from inconsistent coding or their infrequent use outside major neuro-oncology centers. IONM, used to localize and monitor cerebral function, was employed in 46.4% of glioma resections. However, unlike tumor visualization techniques, IONM is primarily used for tumors in proximity to functional brain areas.

In-patient mortality was 6% for all 101,192 in-patient cases involving adult patients diagnosed with malignant glioma [10]. Out of 27,193 hospital cases involving tumor resections, 736 patients died during the same hospital stay, resulting in an in-patient mortality rate of 2.7%. Reported mortality rates after malignant glioma surgeries vary widely across studies, ranging from almost 0 to 8% [2, 6, 7, 13–15, 22]. Factors influencing mortality include case volume, surgeon experience, and patient age, with older age (> 65 years) identified as a predictor for higher mortality [16]. The reasons for increased mortality are multifactorial, including anesthesia-related risks and complications from adjuvant therapies administered during hospitalization. Our analysis showed a higher proportion of patients aged ≥ 65 years among those who died post-surgery compared to survivors. Additionally, fluorescence-guided resection and IONM were significantly less utilized in fatal cases. A causal relationship between these factors and mortality cannot be derived due to the lack of data on important variables such as age, gender, tumor location and size, center size, case load, or team experience. However, the routine use of IONM and tumor visualization techniques in large, experienced centers suggests a potential influence on mortality rates. Further studies should analyze real-world data in more detail to explore this possible relationship.

This study did not analyze supportive and palliative care for malignant glioma patients, who often face significant physical, psychological, social, and spiritual challenges [23]. Current guidelines emphasize early specialized palliative care, with ASCO recommending it within eight weeks of diagnosis for advanced tumor patients [8, 20, 33]. Our previous analysis (2019–2022) demonstrated that only 10.5% of malignant glioma cases in Germany received specialized palliative care, with lower rates in patients under 65 and male patients [10]. Among those who died in the hospital from malignant gliomas, 40.4% received specialized palliative care [10]. In 4.8% of in-patient cases with malignant glioma resection, complex or specialized palliative care was provided, primarily through palliative medicine consultation services.

### Limitations

This study has several limitations:

1. The number of hospital cases does not directly correspond to individual patients.
2. Secondary diagnosis C71 might be coded multiple times within the same case, slightly lowering the actual count. Such instances are likely minimal.
3. Our analysis used the C71 ICD-10-GM code for malignant brain neoplasms, to identify the patient cohort. This code includes a diverse range of WHO 3 and 4 glial tumors not accounting for molecular markers which are as important for prognosis and treatment planning as the grading itself. Moreover, the WHO classification was revised during the study period, and hospitals vary in coding practices [16]. Despite these ambiguities, glioblastoma, IDH-wildtype, WHO grade 4, represents most malignant gliomas. Using ICD-10 coding is standard in health services research, despite its challenges.
4. Treatment guidelines for malignant gliomas consider factors not available in the InEK dataset, such as molecular markers, patient performance scores, tumor characteristics and recurrence, and patient preferences. These unaccounted variables could influence treatment decisions.
5. Data on quality of life are crucial but unavailable in this study design.
6. We examined accompanying diagnoses and complications, but distinguishing symptoms or illnesses caused by the tumor, adjuvant treatment, or surgery was not feasible. Analyzed T80-89 codes cover complications of surgical or medical treatment. We are unsure if all complications and symptoms were accurately coded, possibly underestimating real-world numbers. Our paper probably indicates minimum frequencies of symptoms and complications.
7. The study period spans in part the SARS-CoV-2 pandemic, potentially affecting treatment outcomes. The exact impact cannot be precisely estimated from the data available. These findings likely reflect the COVID-19 phase, marked by limited hospital resources.
8. Hospital mortality was calculated based on hospital cases, but specific causes of death were not specified, which precluded determining neurosurgery-related mortality. Nonetheless, hospital mortality is a relevant parameter for patient advice.
9. We lack data on outpatient care of neuro-oncological patients, less relevant for tumor resections but highly relevant for chemotherapy and radiotherapy, as most treatments are performed in an outpatient setting.
10. The findings' reliability depends on accurate and consistent coding practices in the InEK database. Misclassification or inconsistent coding could introduce biases or inaccuracies. However, prior research has reported high reliability within the German healthcare context [24].
11. Time intervals, such as those between surgery, antitumor therapy, and death, crucial for assessing the quality of oncological treatments, cannot be determined from the InEK data.

## Conclusion

This study offers a comprehensive insight into in-patient surgical treatment for malignant glioma patients in Germany. Over four years, 27,193 cases involved surgical resection, mostly using various modern techniques like microsurgical techniques (95%), intraoperative navigation systems (84%), fluorescence-guided surgeries (45.6%), and IONM (46.4%). The mortality rate was 2.7%, and significantly higher in patients aged 65 and older.

## Supplementary Information

Below is the link to the electronic supplementary material.

Supplementary file1 (JPG 200 kb)

Supplementary file2 (XLSX 13 kb) **Supplementary Table 2** Detailed case numbers and complication data for hospital cases involving malignant glioma patients treated with radiation therapy or medical tumor therapy

Supplementary file3 (XLSX 24 kb) **Supplementary Fig. 1** Hospital cases of neurosurgical tumor resection for malignant gliomas and associated mortality rates from 2019 to 2022. Figure displays the annual case numbers of neurosurgical tumor resections for malignant gliomas (**A**) and the corresponding mortality rates (**B**) from 01/2019 to 12/2022

## Data Availability

No datasets were generated or analysed during the current study.
