# Supplementary figures and images for: In-patient neurosurgical tumor treatments for malignant glioma patients in Germany
Source: J Neurooncol. 2024 Oct 10;170(3):527–42. doi: 10.1007/s11060-024-04784-2 (PMC11615011; doi:10.1007/s11060-024-04784-2)

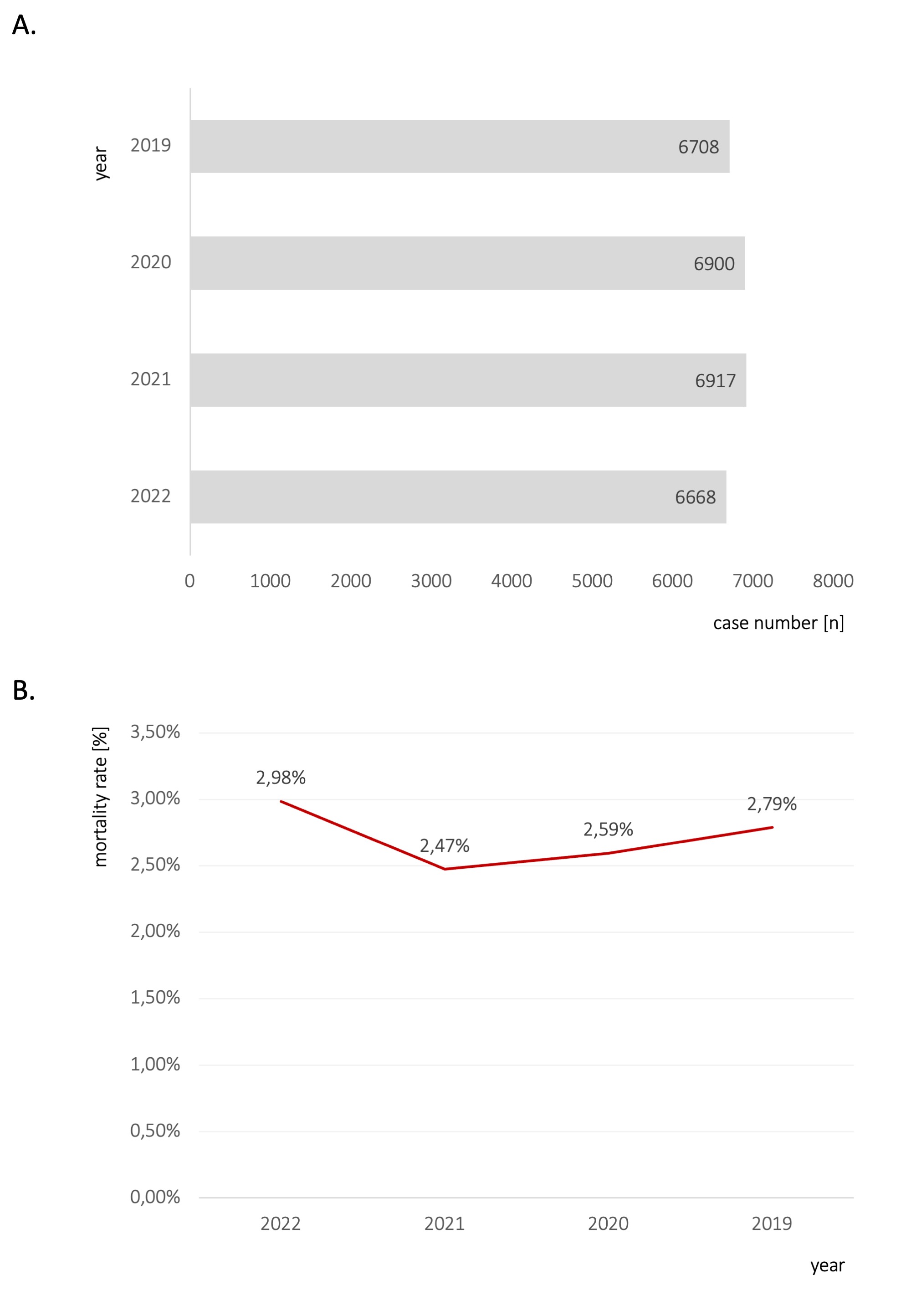

Supplement: Supplementary file 1 — Supplementary file1 (JPG 200 kb) [file 11060_2024_4784_MOESM1_ESM.jpg]
